# IPScan: Detecting novel intronic PolyAdenylation events with RNA-seq data

**DOI:** 10.1371/journal.pcbi.1013668

**Published:** 2025-11-11

**Authors:** Naima Ahmed Fahmi, Sze Cheng, Jeovani Overstreet, Qianqian Song, Jeongsik Yong, Wei Zhang

**Affiliations:** 1 Department of Computer Science, University of Central Florida, Orlando, Florida, United States of America; 2 Department of Biochemistry, Molecular Biology and Biophysics, University of Minnesota Twin Cities, Minneapolis, Minnesota, United States of America; 3 Department of Health Outcomes and Biomedical Informatics, University of Florida, Gainesville, Florida, United States of America; University of Maryland School of Medicine, UNITED STATES OF AMERICA

## Abstract

Intronic PolyAdenylation (IPA) is an important post-transcriptional mechanism that can alter transcript coding potential by truncating translation regions, thereby increasing transcriptome and proteome diversity. This process generates novel protein isoforms with altered peptide sequences, some of which are implicated in disease progression, including cancer. Truncated proteins may lose tumor-suppressive functions, contributing to oncogenesis. Despite advancements in Alternative PolyAdenylation (APA) analysis using RNA-seq, detecting and quantifying novel IPA events remains challenging. To address this, we developed IPScan, a computational pipeline for precise IPA event identification, quantification, and visualization. IPScan has been benchmarked against existing methods using simulated data, different human and mouse cell lines, and TCGA (The Cancer Genome Atlas) breast cancer datasets. Differential IPA events under different biological conditions were quantified and validated via qPCR.

## Author summary

APA affects transcript coding potential by truncating translation regions, thereby increasing transcriptome and proteome diversity. A specific type, IPA, generates alternative protein isoforms. In this study, we introduce IPScan, a newly developed framework for detecting novel IPA events from RNA-seq data, as well as quantifying and visualizing these events. Our evaluation on simulated RNA-seq data, human and mouse cell lines, and real cancer patient data demonstrates that IPScan significantly outperforms state-of-the-art models. Furthermore, we confirm that the detected IPA events are associated with cancer outcomes, offering an additional layer of molecular signatures for downstream analysis.

## Introduction

Alternative PolyAdenylation (APA) is a key post-transcriptional mechanism that regulates gene expression by generating mRNA isoforms with distinct 3′ untranslated regions (3′-UTRs). This process influences mRNA stability, localization, and translation, adding complexity to transcriptome regulation [1,2]. A specialized form of APA, Intronic PolyAdenylation (IPA), occurs within introns and can lead to early transcription termination, producing either truncated coding mRNAs or non-coding transcripts. This mechanism alters protein function by either eliminating essential domains or introducing novel peptide sequences. Dysregulated IPA has been implicated in multiple diseases, including leukemia, multiple myeloma, diabetes, and non-alcoholic fatty liver disease [3–6]. Moreover, IPA-driven premature termination can disrupt tumor suppressors such as *DICER* and *FOXN3* while generating oncogenic isoforms of genes like *CARD11*, *MGA*, and *CHST11* [3]. Additionally, IPA contributes to drug resistance by altering the expression of key genes, such as *TOP2α*, which affects the response to inhibitors in leukemia cell lines (HL-60, CEM, and K562) [7]. The widespread prevalence of IPA, with more than 12,500 genes in the human genome exhibiting annotated intronic APA events [8], underscores its significance in transcriptome regulation and disease pathogenesis. Understanding IPA dynamics is crucial for refining genome annotations and developing predictive models for disease phenotypes.

Leveraging high-throughput RNA-seq technology enables accurate quantification of the transcriptome, providing deeper insights into previously unexplored molecular mechanisms [9,10]. While standard RNA-seq protocols generate sequencing libraries from the entire transcript, 3′-end-seq technology specifically amplifies only the 3′ end of transcripts [11]. This method offers quantitative, genome-wide profiling of the 3′ ends of polyadenylated coding and non-coding transcripts. Typically, peaks in 3′-end-seq data occur upstream of a transcript’s termination point, indicating potential isoform truncation sites [12]. In humans, most polyadenylation sites (polyA sites) are preceded by upstream hexamers (ATTAAA or AATAAA), known as polyadenylation signals (PAS) [13,14]. The presence of 3′-end-seq peaks or PAS within a gene’s coding region can reveal mRNA truncation and potentially lead to the discovery of previously unannotated isoforms and novel protein products.

Several studies have employed RNA-seq, alongside other sequencing protocols, to identify and quantify APA events across various genomic regions. Computational tools such as DaPars [15], APAtrap [16], TAPAS [17], APA-Scan [18], and QAPA [19] are comprehensive and well-established for detecting APA events, though they primarily focus on 3′-UTR APA events. Among more recent studies, IPAFinder [20] targets IPA events by analyzing RNA-seq read coverage fluctuations within intronic regions, while APAlyzer [21] relies on the previously annotated PolyA_DB [22] database to identify and quantify IPA isoforms. APAIQ [23] combines DNA sequence information with RNA-seq read coverage to detect APA sites but is not specifically designed for IPA. InPACT [24], a newer IPA detection method, uses a convolutional neural network module to identify potential polyA sites and a read module to identify unannotated terminal exons.

In this study, we introduce IPScan, a tool that integrates RNA-seq read coverage data with 3′-end-seq peaks to identify novel IPA events and validate detected sites using wet-lab qPCR experiments and PacBio Iso-Seq long-read sequencing data. Tests on both simulated datasets and real human and mouse cell lines demonstrate IPScan’s ability to detect differential IPA events across diverse biological conditions. Furthermore, analysis of The Cancer Genome Atlas (TCGA) breast cancer (BRCA) dataset [25] reveals associations between IPScan-identified IPA events and potential biomarkers relevant to tumorigenesis.

## Results

**Fig 1:**
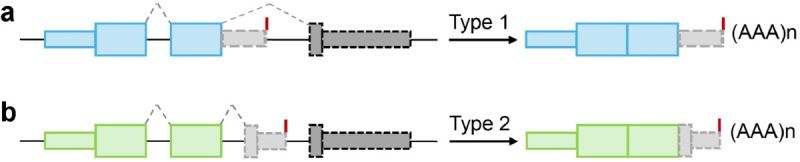
Two types of IPA events. a) Type 1: Occurs when the first step of splicing is inhibited, resulting in polyadenylation within the downstream intron. b) Type 2: Involves inclusion of a cryptic exon within the downstream intron, which serves as the new 3’-end exon. Introns are shown as black solid lines, and polyA sites are indicated by red vertical lines.

IPScan identifies two types of IPA events: Type 1 and Type 2, as shown in Fig 1. In Type 1 events, polyadenylation occurs within a downstream intron, resulting in a hybrid exon-intron structure that serves as the 3′-end exon. In Type 2 events, a cryptic exon within the downstream intron is recognized and used as the 3′-end exon.

**Fig 2:**
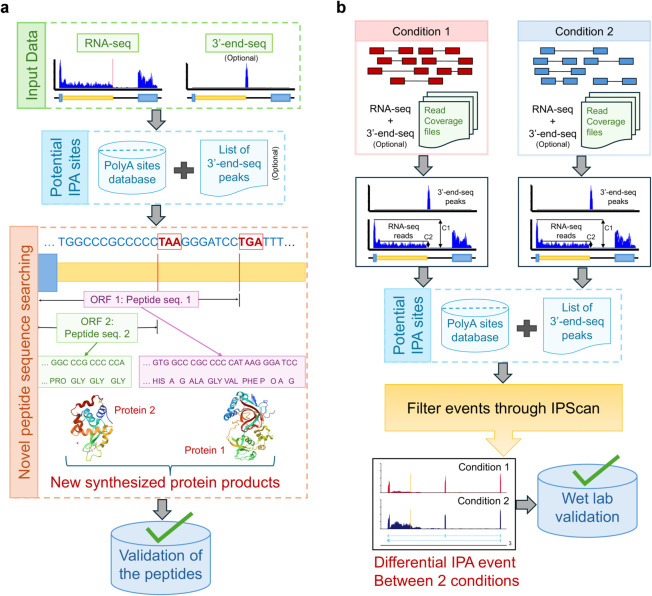
Workflow of IPScan. (a) Schematic overview of IPScan’s process for detecting novel IPA events and generating peptide sequences from newly identified truncated isoforms. (b) Diagram illustrating the detection and quantification of differential IPA events between two conditions, with coverage plots depicting read coverage flanking the IPA sites in both conditions.

Moreover, IPScan is designed to handle two key scenarios. First, it detects novel IPA events present in the input sample (Fig 2a). Second, it quantifies differential IPA site usage by comparing two distinct biological conditions (Fig 2b). To evaluate its performance, we applied IPScan to multiple simulated and real datasets, including human and mouse cell lines as well as TCGA breast cancer patient samples. Our analysis uncovered several unannotated IPA sites, underscoring their potential for inclusion in protein annotation databases.

### Experimental results with simulated RNA-seq data

In our simulation experiment, we generated synthetic RNA-seq samples with sequencing depths ranging from 5 million (M) to 50M short reads using Flux-Simulator [26]. To model IPA events, we introduced 1,000 synthetic events per sample, consisting of 500 Type 1 and 500 Type 2 events. Further details of this process are provided in the section “Materials and Methods”. To simulate dynamic changes in IPA events under different biological conditions, we generated paired samples (control vs. case), each containing 50M reads and designed to include 1,000 synthetic differential IPA events to assess IPScan’s ability to detect differential IPA usage. For each condition, three technical replicates with varied parameter settings were created using Flux-Simulator.

#### IPScan is robust across varying sequencing depths.

**Fig 3:**
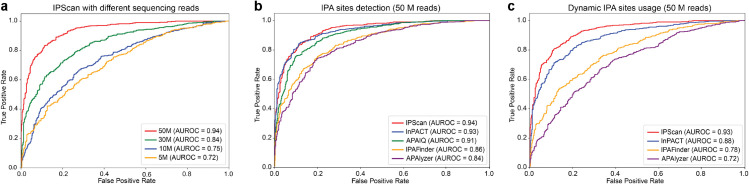
AUC plots illustrating the performance of IPScan. (a) Simulation experiment evaluating IPScan at different sequencing depths. ROC curves show detection accuracy across RNA-seq read depths ranging from 5M to 50M. (b–c) Comparison of IPScan with four baseline methods: (b) identification of potential IPA sites and (c) detection of differential IPA events between conditions. In both analyses, performance was benchmarked against 1,000 simulated ground-truth events.

IPScan demonstrates high sensitivity, detecting IPA events even at low read coverage. Among the 1,000 simulated IPA events, it successfully identified 609 (61%), 699 (70%), 852 (85%), and 918 (92%) at sequencing depths of 5M, 10M, 30M, and 50M reads, respectively. As shown in Fig 3a, while detection performance improves with increasing sequencing depth, IPScan maintained a comparatively high AUC score of 0.72 even at the lowest tested coverage (5M reads), underscoring its robustness in detecting IPA events across a wide range of sequencing depths.

#### IPScan outperforms the baselines.

To evaluate the accuracy of IPA event detection and the estimation of dynamic usage across conditions, we compared IPScan with four state-of-the-art computational tools: IPAFinder, APAlyzer, InPACT, and APAIQ. On the simulated RNA-seq dataset with 50M reads, IPScan demonstrated superior precision and accuracy in IPA site detection (Fig 3b). Although InPACT and APAIQ performed comparably, IPScan achieved the highest AUC score of 0.94, outperforming all other baselines. At varying read depths (30M, 10M, and 5M), IPScan achieved top or near-top performance, with the corresponding AUROC plots shown in Figs A–C in *S1 Appendix*.

Among the 1,000 synthetic differential IPA events between control and case samples, IPScan correctly identified 897 as significantly different between conditions, surpassing InPACT (870), IPAFinder (801), and APAlyzer (648). APAIQ was not included in this comparison because it is not designed to evaluate condition-specific changes in IPA site usage. As shown in Fig 3c, AUC scores were calculated for paired samples with 50M reads per condition. IPScan achieved the highest AUC (0.93), exceeding InPACT (0.88), while IPAFinder and APAlyzer exhibited lower values. For each condition, three technical replicates were included, and significance was determined using a Wilcoxon rank-sum test (*p*-value <0.05) together with truncation ratio (TR) difference between conditions ($|TR_{1}-TR_{2}|>0.2$).

Beyond accuracy, false positive rates varied among methods. APAlyzer reported the largest number of significant events, but only 70% were true IPA events, indicating a high false positive rate. IPAFinder performed better, with a 19% false positive rate, whereas IPScan achieved the lowest rate at 13.6%. Together, these results highlight IPScan’s superior detection accuracy and robustness for identifying differential IPA events across diverse biological conditions.

### IPA profiling across human and mouse cell lines

To further evaluate IPScan’s performance, we applied it to various mammalian cell types, including Tsc1^−/−^ mouse embryonic fibroblasts (MEFs) and wild-type (WT) MEFs, as well as the human breast cancer cell lines MCF7 and BT549. In both breast cancer cell lines, we analyzed samples treated with DMSO (mock) or Torin 1, a potent mTOR inhibitor.

#### IPScan detects novel IPA events.

**Fig 4:**
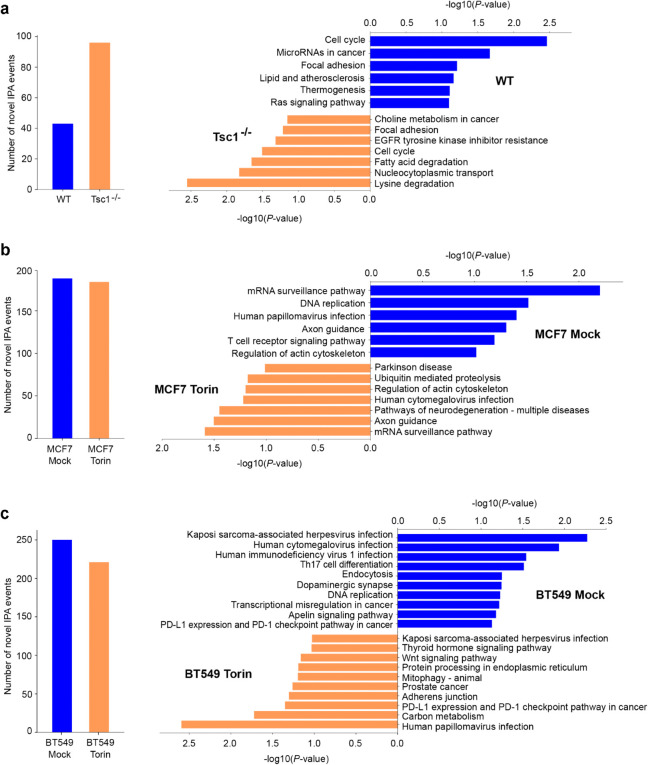
Novel IPA events and associated KEGG pathway enrichment analysis. The vertical bar plots (left) depict the number of novel IPA events identified by IPScan, while the horizontal bar plots (right) present KEGG pathway enrichment results for (a) mouse MEF cell lines and (b, c) breast cancer cell lines. The x-axis indicates the *p*-value (-log10 normalized), and the y-axis lists the corresponding KEGG pathways. Pathways enriched by genes with higher expression in the first group are shown at the top, whereas those enriched in the second group appear at the bottom.

IPScan successfully identified novel IPA sites across both human and mouse cell lines. As shown in Fig 4 (left panels), the number of detected IPA events varied across samples. In human cell lines, Torin-treated samples exhibited a higher number of IPA events compared to mock-treated ones. Modulations in mTOR activity within cellular systems result in a bifurcated expression profile of IPA. Therefore, the results explain well the identification of novel IPAs in cells treated with Torin 1 [8].

The detected IPA events were further classified into two types: Type 1, where splicing inhibition leads to polyadenylation within a downstream intron, and Type 2, where a cryptic exon within the downstream intron becomes the new 3’-end exon (Fig 1). As summarized in Table 1, IPScan detected a significant number of *novel* Type 1 events in all samples, while Type 2 events were less frequent. This suggests that mTOR pathway perturbation primarily affects splicing dynamics and promotes premature polyadenylation within introns (Type 1) rather than cryptic exon inclusion (Type 2).

**Table 1:** Total number of IPA events detected by IPScan for each cell line, categorized by type. Events are classified into Type 1 (polyadenylation within a downstream intron due to splicing inhibition) and Type 2 (cryptic exon inclusion forming a new 3’-end exon).

Sample	IPA events	Type1	Type2
MEF WT	43	30	13
MEF Tsc1^−/−^	96	81	15
MCF7 Mock	188	184	4
MCF7 Torin	184	182	2
BT549 Mock	250	242	8
BT549 Torin	221	202	19

**Fig 5:**
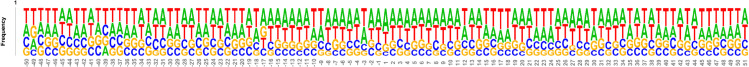
Nucleotide composition surrounding IPA sites detected by IPScan in the Mouse Tsc1^−/−^ MEF sample. The x-axis indicates positions relative to the IPA sites (±50 bp), and the y-axis shows the frequency of each nucleotide at each position.

To validate the predicted IPA sites, we analyzed the nucleotide composition surrounding these regions. Polyadenylation is typically driven by PAS, such as ATTAAA, AATAAA, or single nucleotide variants, located upstream of polyA sites to signal transcript termination. In the Mouse Tsc1^−/−^ MEF sample, the nucleotide profile around IPA sites identified by IPScan showed a significant enrichment of A and T nucleotides in upstream regions, supporting the potential for isoform truncation (Fig 5). This AT-rich signature aligns with known PASs, reinforcing the accuracy of IPA site detection. Similarly, nucleotide distribution analysis in human cell lines revealed a higher frequency of A and T bases surrounding the detected IPA sites (Figs D–H in *S1 Appendix*).

Pathway analysis is essential to understand gene function and the broader biological significance of detected IPA events. After identifying genes affected by IPA, we performed KEGG pathway enrichment analysis to examine their impact on biological processes. As shown in Fig 4 (right panels), the enriched KEGG pathways for IPA events detected by IPScan vary across the three cell lines analyzed in this study. The results indicate that mouse and human samples with different mTOR activation levels are involved in key cellular processes associated with cancer hallmarks [27]. Notable pathways include the cell cycle, focal adhesion, Ras signaling, and PD-L1 expression and PD-1 checkpoint pathways, all of which play critical roles in cancer progression [27]. Interestingly, the enriched pathways exhibit species- and cell line-specific patterns, suggesting that the biological functions of these novel IPA genes may depend on cellular context and influence breast cancer aggressiveness. Additionally, pathways such as mRNA surveillance, axon guidance, and actin cytoskeleton regulation were consistently observed among both upregulated and downregulated IPA genes when comparing samples with high and low mTOR activation. These findings underscore the significance of mTOR-regulated pathways in tumorigenesis, particularly through IPA regulation.

To independently validate the novel IPA isoforms identified in our study, we leveraged publicly available long-read transcriptomic data. Long-read sequencing technologies, such as PacBio single-molecule real-time (SMRT) Iso-Seq [28], sequence entire transcripts end to end without requiring computational assembly or inference [29]. By capturing complete cDNA molecules without fragmentation, Iso-Seq provides an orthogonal layer of evidence free from read-coverage biases and assembly heuristics, making it particularly well suited for validating candidate IPA isoforms predicted from short-read data [30,31].

**Fig 6:**
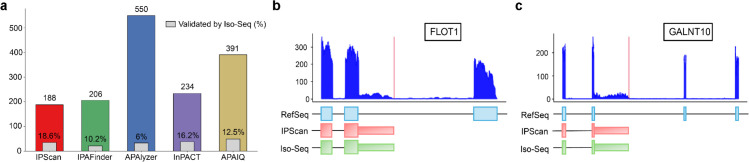
Experimental validation of two IPA events detected exclusively by IPScan using the PacBio Iso-seq platform. (a) Ratio of IPA events detected by each method that were validated by Iso-Seq data. (b–c) Two RefSeq unannotated isoforms from (b) *FLOT1* and (c) *GALNT10*, identified only by IPScan, are supported by long-read Iso-Seq annotations and corresponding read-coverage plots.

Using Iso-Seq as an additional benchmark, we found that approximately 18.6% of the novel IPA isoforms predicted by IPScan were corroborated by full-length long-read transcripts. By comparison, IPAFinder, APAlyzer, InPACT, and APAIQ achieved lower validation rates of 10.19%, 6 %, 16.2%, and 12.5%, respectively (Fig 6a). These results highlight IPScan’s superior accuracy in capturing bona fide IPA isoforms, particularly those overlooked by methods that rely more heavily on pre-annotated polyA sites. Notably, among the events exclusively detected by IPScan, two isoforms derived from *FLOT1* and *GALNT10* were supported by Iso-Seq data (Fig 6b–6c). Both of these isoforms are novel with respect to current RefSeq annotations. The ability of Iso-Seq to recover these unannotated transcripts provides strong, independent confirmation of IPScan’s predictions.

#### IPScan measures the differential usage of IPA events.

**Fig 7:**
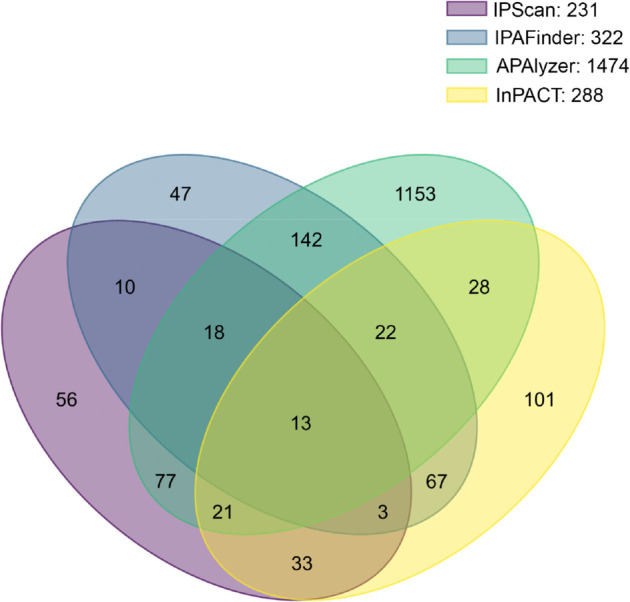
Venn diagram illustrating the overlap of differential IPA events detected in WT and Tsc1^−/−^ samples by four methods: IPScan, IPAFinder, APAlyzer and InPACT. The diagram highlights shared and unique IPA events identified by each method.

Using IPScan, we identified widespread differential usage of novel IPA sites across various conditions in human and mouse cell lines. Detection was based on a significance threshold defined by a chi-squared test (*p*-value <0.05) and a TR difference between conditions ($|TR_{1}-TR_{2}|>0.2$). In the absence of ground truth data for real experimental samples, we evaluated IPScan’s performance by assessing its concordance with baseline methods. Unlike IPAFinder, which detects both annotated and novel events by leveraging read-coverage fluctuations, APAlyzer primarily captures expression changes between regions upstream and downstream of known IPA sites and depends on a pre-annotated database (polyA_DB) [22]. To quantify the overlap of differential IPA events identified by IPScan and these tools, we constructed a four-set Venn diagram comparing IPScan, IPAFinder, APAlyzer, and InPACT in mouse WT vs. Tsc1^−/−^ MEF samples (Fig 7). APAIQ was not included in this comparison, as it is not designed to evaluate condition-specific changes in IPA site usage. Among the methods, APAlyzer reported the largest number of differential events, largely due to its reliance on pre-annotated polyA sites. Notably, the limited overlap among the four methods underscores the novelty of IPScan’s findings.

**Fig 8:**
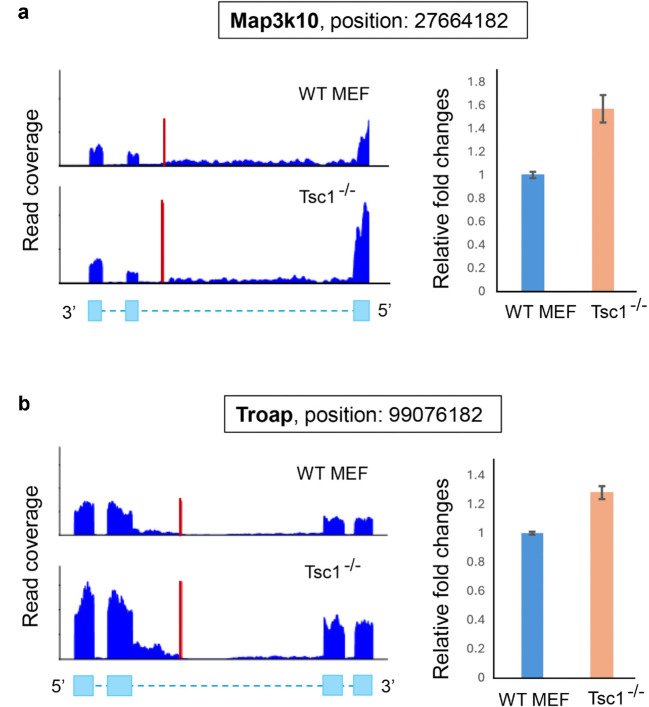
Experimental validation of two IPA events detected exclusively by IPScan. Quantitative PCR (qPCR) analysis shows significantly elevated expression of *MAP3K10* and *TROAP* transcripts in Tsc1^−/−^ MEF cells compared with WT controls, supporting the activation of novel IPA sites in these genes.

Among the 231 differential IPA events detected by IPScan, 56 events were exclusively detected only by IPScan. We further validated two of these unique events, *MAP3K10* and *TROAP*, through qPCR and alignment plot analyses. The qPCR results confirmed increased expression of the truncated isoforms in Tsc1^−/−^ compared to WT, consistent with IPScan’s predictions (Fig 8). Details of the qPCR experiments and primer design are provided in the *S1 Appendix*.

### IPA analysis in TCGA breast cancer data

IPScan was applied to 1,219 TCGA breast cancer (BRCA) samples [25]. To assess differential IPA usage between tumor and normal tissues, we analyzed 1,106 tumor samples and 113 normal samples. Clinical data for 1,108 BRCA patients were obtained from cBioPortal [32].

#### IPScan identifies differential IPA usage in breast cancer tumor-normal samples.

**Fig 9:**
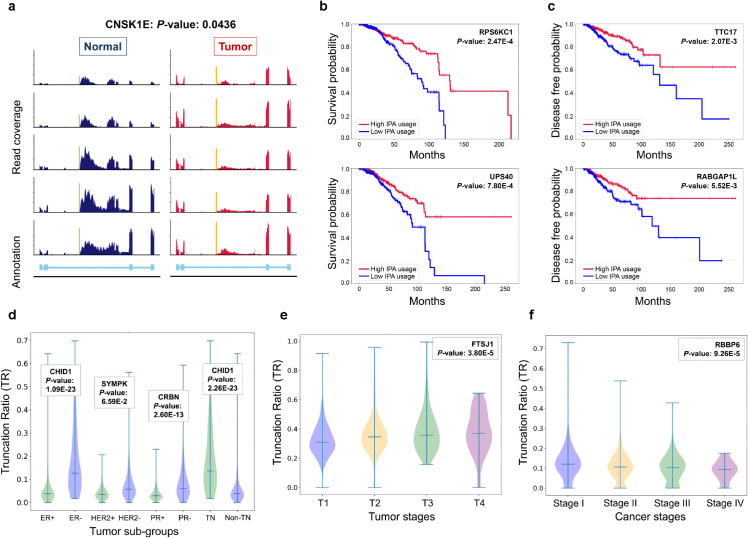
IPScan analysis of TCGA BRCA data. (a) Validation of an IPA event detected by IPScan (*CSNK1E*: Chr22:38296209) using read-coverage plots from five normal and five tumor samples. The x-axis indicates genomic position, and the y-axis shows read coverage; gene annotation is provided in the bottom panel. (b–c) Kaplan–Meier plots showing associations between IPScan truncation ratio (TR) values and (b) overall survival or (c) disease-free rate for two representative IPA events. (d) Significant IPA events across tumor subtypes (ER, PR, HER2, and triple-negative [TN]); the significant event for each subtype is shown separately for receptor-positive and receptor-negative samples. (e) Boxplots of IPScan TR values for an IPA event in *FTSJ1* across four tumor stages. (f) Boxplots of IPScan TR values for an IPA event in *RBBP6* across four tumor stages.

Using IPScan, we identified a substantial number of IPA events that exhibited significant differences in site usage between breast cancer tumor and normal samples. These findings suggest that differential IPA usage is linked to cancer-specific regulatory mechanisms, as IPA events influence mRNA stability, translation, and protein production. Such dynamic alterations in IPA usage highlight their potential as biomarkers for cancer diagnosis or prognosis, offering valuable insights into the molecular mechanisms driving tumor progression. As illustrated in Fig 9a, an alignment plot demonstrates the read coverage of a novel IPA event across multiple samples, showing a clear separation between tumor and normal samples based on TR values. The statistical significance of this separation was validated using an unpaired t-test (*p*-value < 0.05).

#### IPScan-detected IPA events associated with clinical variables.

We analyzed the associations between IPScan-derived TR values and various clinical variables. Specifically, we evaluated the relationship between IPA site usage and both overall survival and disease-free rates. The analysis focused on a selected set of IPA events from tumor samples, prioritizing genes with high expression levels to reduce ambiguity. Kaplan–Meier survival plots revealed significant correlations between IPA usage and survival outcomes (Fig 9b) as well as disease-free rates (Fig 9c). These findings were further supported by log-rank tests, which demonstrated statistical significance based on the corresponding *p*-values.

We also examined IPA patterns, measured as truncation ratio (TR) values, across hormone receptor subtypes in breast cancer tissues. These subtypes—Estrogen Receptor positive/negative (ER+/ER-), Progesterone Receptor positive/negative (PR+/PR-), Human Epidermal Growth Factor Receptor 2 positive/negative (HER2+/HER2-), and Triple-Negative (TN/non-TN)—play critical roles in cancer progression. TR values from IPScan analysis revealed clear distinctions between receptor-positive and receptor-negative subgroups (Fig 9d), providing insights that may inform treatment strategies. In addition, TR values showed significant differences across the four stages of cancer progression and tumor development (Fig 9e, 9f). These patterns highlight the potential of IPScan-derived IPA events as robust indicators for disease staging and prognosis.

#### IPScan identifies novel peptide sequences in breast cancer patient samples.

From 1,219 TCGA BRCA samples, IPScan identified 3,019 IPA events. Among these, 1,315 unique unannotated peptide sequences were predicted based on the open reading frames (ORFs) of upstream annotated exons. To validate these peptides, we examined their overlap with Pfam domains affected by IPA events. Our analysis revealed that 535 IPA events resulted in the loss of functional Pfam domains due to missing peptides within these regions. For example, an IPA event in CASP10 (caspase-10) leads to loss of the *peptidase_C14* domain while retaining the DED (death effector domain), potentially generating truncated CASP10 isoforms that function independently of canonical death-signal activation [33,34]. These findings suggest that protein isoforms arising from IPA events may gain deregulated or novel functions compared with their full-length counterparts, thereby reshaping the functional proteome.

Additionally, we applied PepQuery [35], a tool designed to identify known and novel peptides in local or publicly available mass spectrometry-based proteomics datasets. Cross-referencing two proteomics datasets, “TCGA Breast Cancer Proteome PDC000173” and “TCGA Breast Cancer Phospho-Proteome PDC000173”, PepQuery found no overlap between the novel peptide sequences identified by IPScan and annotated databases. This indicates that these sequences were previously undocumented in existing proteomic resources. Detailed results of the PepQuery analysis are provided in the S1 Appendix.

## Discussion

In this study, we introduced IPScan, a computational tool designed to identify novel IPA sites from RNA-seq data and to quantify dynamic IPA changes under different conditions. IPScan’s performance was validated through multiple layers of experimental evaluation, including comparisons with four state-of-the-art methods—IPAFinder, APAlyzer, InPACT, and APAIQ. Our results demonstrate that IPScan outperforms these methods in accurately and precisely identifying IPA events. Notably, two novel IPA isoforms in *FLOT1* and *GALNT10*, detected exclusively by IPScan, were independently validated using PacBio Iso-Seq long-read sequencing, providing strong orthogonal evidence for their authenticity. Evaluations on simulated RNA-seq data showed that IPScan more effectively predicts IPA isoforms and quantifies dynamic IPA site usage across conditions compared with baseline methods. Importantly, IPScan maintained robust performance even in low-coverage samples, achieving moderate to high accuracy across varying sequencing depths. Furthermore, it exhibited the highest precision and the lowest false positive rates among all tested methods. To further assess its practical utility, we applied IPScan and the baseline tools to human and mouse cell lines. While IPScan consistently reported the lowest false positive rates in simulated datasets, it also identified differential IPA events that were undetected by the baseline methods in cell line data. Two such events, involving *MAP3K10* and *TROAP*, were validated through wet-lab qPCR experiments, confirming their differential expression and supporting the accuracy of IPScan’s predictions. These findings establish IPScan as a robust and reliable tool for IPA detection, offering significant improvements over existing methods.

IPA events are frequently observed in breast cancer [36]. Using IPScan, we analyzed 1,219 TCGA breast cancer samples and identified significant alterations in IPA events between tumor and normal samples, including those in key cancer driver genes. Novel IPA events were detected in oncogenes such as *GNB1*, *SMARCE1*, *STAT6*, *CUL4A*, and *IQGAP1* [37]. Additionally, dynamic IPA site usage was observed in well-known tumor suppressor genes, including *TSC1*, *HSD17B2*, *CD58*, *RUNX1* and *INPP4B* [38]. In the case of the transcription factor *RUNX1*, the IPA event results in truncation within the Runt domain (an evolutionarily conserved protein domain), rendering this domain non-functional and potentially disrupting the critical dimerization activity required for *RUNX1* function. Under normal conditions, *RUNX1* is robustly expressed in both luminal and basal cells of breast tissue [39,40]. In contrast, many breast cancers—particularly aggressive subtypes—exhibit reduced or absent *RUNX1* expression [39,41]. Functionally, *RUNX1* helps preserve the epithelial phenotype by promoting E-cadherin expression, thereby supporting cell–cell adhesion and suppressing migratory behavior. When epithelial–mesenchymal transition (EMT) is induced (e.g., by TGF-*β* signaling or serum deprivation), *RUNX1* expression declines, leading to increased mesenchymal marker expression and enhanced invasiveness [39]. Together, these observations suggest that IPA-mediated disruption of *RUNX1* introduces previously unrecognized pathogenic mechanisms that may contribute to breast cancer progression.

Further investigation into the biological functions of the differentially expressed IPA genes in TCGA breast cancer samples, particularly those regulated by mTOR signaling, revealed their involvement in critical oncogenic pathways. For instance, dysregulation in axon guidance pathways, which are known to promote cell proliferation and adhesion in cancer, was identified [42]. Additionally, we observed disruptions in the PD-1/PD-L1 immune checkpoint pathway, a mechanism frequently exploited by cancer cells to evade immune surveillance [43]. As IPA events often produce truncated protein products with altered functions, the newly identified unannotated IPA proteins regulated by mTOR signaling may contribute to breast cancer progression. These proteins likely dysregulate axon guidance and immune surveillance pathways, potentially promoting tumor growth and reducing the clinical efficacy of PD-1/PD-L1 inhibitors in breast cancer patients. These findings highlight the importance of IPA in understanding breast cancer biology and improving therapeutic strategies.

IPScan offers broader and more comprehensive annotations than existing methods. Despite these strengths, several areas remain for future improvement. For example, the optional use of matched 3’-end-seq data, while clearly indicated as an “optional” input, may need further consideration given the limited availability of datasets that include both matched 3’-end-seq and RNA-seq. In practice, however, 3’-end-seq data do not have to be perfectly matched to every RNA-seq sample. 3’-end-seq data derived from the same tissue type, disease context, or well-characterized cell lines can provide valuable reference information for annotating and validating polyA sites. This flexibility helps extend IPScan’s applicability even when fully matched datasets are not available.

In addition, IPScan’s current implementation relies on read-coverage-based quantification of truncation ratios and is therefore not directly applicable to single-cell RNA-seq analysis. Unlike bulk RNA-seq, single-cell RNA-seq typically suffers from lower sequencing depth, higher technical noise, and substantial dropout rates, all of which hinder the accurate detection of fine-scale read-coverage patterns needed to identify IPA events. As part of our future work, we plan to extend IPScan to single-cell resolution while addressing these challenges. One promising direction is to integrate PacBio long-read sequencing with single-cell RNA-seq to improve isoform-level resolution and enhance the accuracy of IPA event detection. In addition to detection accuracy, the computational cost of IPA analysis tools is an important consideration for large-scale studies. Our benchmarking shows that runtimes vary widely across methods, from less than an hour for APAlyzer to several hours on CPU-based systems for IPAFinder and InPACT, with deep learning-based approaches such as APAIQ requiring even longer CPU runtimes but benefiting from significant GPU acceleration. Relative to these tools, IPScan exhibits competitive runtime performance. On a standard compute node equipped with 16 CPU cores (Intel(R) Xeon(R) CPU E5-2620 v4 @ 2.10GHz, 64-bit) and 125 GB RAM, the complete analysis of an RNA-seq dataset containing ∼50 million paired-end aligned reads required about 80 minutes using IPScan. Notably, extraction of peaks from the 3’-end-seq data and generation of the merged database accounted for nearly 30 % of the total runtime. Once a pre-generated list of potential IPA events is available, IPScan runs substantially faster when applied to large-scale datasets such as TCGA BRCA, requiring only ∼40 minutes per sample. These results indicate that, while not the fastest among all benchmarks, IPScan provides a practical balance between computational cost and analytical depth, making it well suited for large-scale transcriptomic analyses.

## Materials and methods

### Data preparation

**Simulation data.** In our experiments, we used Flux Simulator [26] to generate paired-end short reads and simulate *in silico* RNA-seq experiments based on a ground truth transcript expression profile and IPA events. Specifically, we generated four sets of synthetic RNA-seq samples with sequencing depths of 5M, 10M, 30M, and 50M reads, with 500 Type-1 and 500 Type-2 events on each sample. To assess dynamic IPA usage under different conditions, we prepared paired control and case samples with 50M reads each. For all simulated samples, gene expression levels were assigned to follow a Poisson distribution to mimic real RNA-seq data. To ensure accurate IPA detection and minimize ambiguity from low-expression genes, IPA events were simulated only in highly expressed genes. Each IPA site was modeled with a short and long isoform pair, where the IPA site represents the endpoint of the unannotated short isoform, while the original full-length isoform serves as the long isoform. Genomic locations, including intron-exon structures, were extracted from RefSeq annotations [44]. The specific parameter configurations are provided in Table A in *S1 Appendix*. All simulated RNA-seq samples were aligned to the reference genome using HISAT2 [45] and converted into read-coverage files using SAMtools [46]. This pipeline ensured high-quality simulation and alignment, allowing for a robust evaluation of dynamic IPA usage.

**Cell-line data.** To conduct our experiments on real tissue samples, we evaluated different sets of human and mouse cell lines [12,47,48] using IPScan. Analyses were performed on both RNA-seq and matched 3′-end-seq datasets for each sample. For mouse embryonic fibroblast (MEF) RNA-seq samples, we analyzed poly(A^ + ^) RNAs isolated from Tsc1^−/−^ and wild-type (WT) MEFs. The RNA-seq analysis generated 63,742,790 paired-end reads for WT and 74,251,891 paired-end reads for Tsc1^−/−^ MEFs using the HiSeq platform, with each read being 50 base pairs long. Short reads were aligned to the mm10 reference genome using HISAT2. For the 3′-end-seq analysis, reads from WT and Tsc1^−/−^ MEFs were preprocessed by trimming polyA tails from the 3′-ends and filtering out low-quality reads (Phred score < 30) or reads shorter than 25 base pairs. The remaining reads were aligned to the mouse mm10 reference genome using Bowtie2 [49], with no mismatches allowed. In total, 6,186,893 reads were aligned for WT samples, and 5,382,111 reads were aligned for Tsc1^−/−^ samples. For MEF data analysis, 24,511 IPA sites were annotated by overlapping 27,091 UCSC polyA sites from the mouse mm10 genome with 65,488 3′-end peaks identified from the 3′-end-seq MEF samples.

The human cell line data included two sets of Mock vs. Torin-treated samples: MCF7 Mock vs. Torin and BT549 Mock vs. Torin. For MCF7, the RNA-seq data generated 115,450,097 paired-end reads for Mock and 108,176,924 paired-end reads for Torin-treated samples. In the 3′-end-seq analysis, 4,355,286 reads were mapped to the human hg38 reference genome for MCF7 Mock, and 3,343,638 reads were mapped for MCF7 Torin. Similarly, for BT549, the RNA-seq data produced 131,955,082 paired-end reads for Mock and 138,127,113 paired-end reads for Torin-treated samples. The mapped 3′-end reads totaled 3,348,578 for BT549 Mock and 4,386,406 for BT549 Torin. The same pipeline used for the MEF data was applied to generate read coverage files for the human cell line data. To annotate IPA locations in human breast cancer samples, we identified 20,868 potential IPA sites by intersecting 42,615 UCSC polyA sites from the human hg38 genome with 66,617 3’-end peaks derived from the combined MCF7 and BT549 cell line data.

Human MCF7 Iso-Seq data (SRA: SRX7505753; GEO accession: GSM4251592) were downloaded from the GEO database and consist of single-end PacBio RS II reads from three biological replicates. Raw FASTQ files were aligned to the human reference genome using minimap2 [50] with default Iso-Seq settings, achieving an alignment rate of approximately 91%. The aligned transcripts were then processed with SQANTI3 [51], which integrates long-read isoform evidence with reference annotations to generate an updated transcriptome annotation (GTF) file. SQANTI3 classified the transcripts as either known isoforms (matching reference annotations) or novel isoforms (containing unannotated splice junctions, alternative transcription start or end sites, or intronic polyadenylation events).

**TCGA breast cancer data.** A total of 1,219 breast cancer samples were downloaded from TCGA to investigate the association of IPA events with cancer patient data. The dataset included 1,106 TCGA BRCA tumor samples and 113 normal tissue samples. All samples were aligned to the reference genome, and read coverage files were generated using HISAT2 and SAMtools. Clinical data for the 1,106 BRCA patients were obtained from cBioPortal [32]. While TCGA provides extensive RNA-seq data across multiple cancer types, it does not include 3′-end-seq data. To address this limitation, we utilized the 20,868 potential IPA sites previously identified in human breast cancer cell line data for the analysis of TCGA BRCA samples.

### IPScan workflow

IPScan is designed to discover novel IPA events through two complementary approaches. First, it identifies novel IPA sites and reconstructs the resulting unannotated peptide sequences. Second, it quantifies changes in IPA usage across biological conditions and evaluates their statistical significance. To broaden the search space, IPScan integrates UCSC-annotated polyA sites (PolyA_DB 3) [22] with 3’-end-seq peaks (optional input), generating an extensive catalog of candidate sites. When matched 3’-end-seq data are not available, the method instead leverages existing polyA site databases and performs genome-wide scans for canonical PASs, such as AATAAA and its variants, to predict sites. Candidate IPA events are then refined by focusing on independent introns, introns that do not overlap with any exon from any isoform of the gene, ensuring that only true intronic sites are retained for downstream analyses. This restriction reduces ambiguity arising from alternative splicing and annotated transcript ends, thereby improving both the specificity and reliability of IPA detection.

If a polyA site is present in the nearby region, the read coverage distribution is likely to exhibit a significant drop near the site. Based on this principle, IPScan calculates the TR for each novel event as TR = $\frac{C_{2}}{C_{1}}$ where *C*_2_ represents the average read coverage in the intron region upstream of the IPA site, and *C*_1_ denotes the average read coverage of all the exons of the gene. The detected events are categorized into Type 1 and Type 2 based on their structural formation (Fig 1). If no Type 1 event is observed in the designated region, IPScan searches for Type 2 events, where a cryptic exon serves as the new 3′-end exon, which is entirely composed of intronic sequences (Fig 1). The cryptic exon boundary is defined when the average read coverage of that region reaches at least 80% of the average read coverage of all exons of the gene. This allows for a refined estimation of both the average read depth across all exons (*C*_1_) and the read coverage of the intronic region within the IPA site and the 3′-end exon margin (*C*_2_).

The process involves parameter tuning to enhance the identification of IPA events. For TR = $\frac{C_{2}}{C_{1}}$, events with TR < 0.2 are filtered out. Additionally, a threshold of *C*_2_ > 10 is applied to remove a large portion of false events. For each identified position, peptide sequences are generated based on the Open Reading Frames (ORFs) of the upstream annotated exons. Codons are translated up to the first available stop codon within the given range. For Type 2 events occurring in the intronic region, all three possible ORFs from the tentative start position of the cryptic exon are considered when generating the peptide sequences.

To assess dynamic IPA changes between conditions, IPScan identifies differential events based on differences in TR values. A change in TR for a gene between samples or groups is reported as a differential IPA event. The significance of these events is evaluated using a chi-squared test with a threshold of *p*-value < 0.05 and a TR difference greater than 0.2. For samples with replicates, dynamic IPA usage is assessed using the Wilcoxon rank-sum test, with significance defined as *p*-value < 0.05 and an average TR difference greater than 0.2.

### Implementation

IPScan is implemented as a command-line tool using Python3 scripts. It requires RNA-seq data in BAM format as input. To identify IPA events, IPScan operates in two modules: ‘single’ and ‘differential’. The ‘single’ module detects potential novel IPA sites along with *de novo* peptide sequences, while the ‘differential’ module compares IPA events between two biological conditions. The current implementation supports and has been tested against both human (hg38) and mouse (mm10) genomes using RefSeq annotation. IPScan is easily adaptable to other species when provided with proper annotations and required input files. For graphical visualization, IPScan generates read coverage and annotation plots for user-specified regions. Detailed implementation and execution instructions can be found at github/IPScan.

### Baselines and evaluation methods

In this study, four widely used IPA identification approaches, InPACT, APAIQ, IPAFinder and APAlyzer, were applied to evaluate the performance of IPScan. InPACT characterizes IPA events by reporting the parameters ‘PolyAsite’ and ‘IPA usage’. For APAIQ, candidate IPA events were derived from the set of detected PASs. IPAFinder identifies potential IPA sites through the annotation of ‘IPA_terminal_region’, whereas APAlyzer directly provides the PAS identifier together with its genomic position. To ensure a consistent and unbiased comparison, all detected events from these methods were evaluated against the ground truth within ±50 bp from the reference site.

To evaluate changes in IPA site usage between simulated case and control samples (three replicates per condition), we combined InPACT with DRIMSeq [52] to conduct differential transcript usage analysis, as recommended in the InPACT manuscript [24]. While InPACT itself is not inherently designed for differential comparisons, its output can be coupled with DRIMSeq to enable such analyses. DRIMSeq uses a Dirichlet-multinomial model to assess isoform usage and enables detection of condition-dependent changes in the IPA sites with the criteria of $\left|\log_{2}(\text{Fold Change})\right|$ > 1 and FDR < 0.05. Because APAIQ does not support case–control designs or provide a framework for differential usage testing, it was excluded from this comparison. IPAFinder used a DEXSeq-based statistical approach to determine the dynamic usage of IPA sites. The selection criteria were set to an FDR-adjusted *p*-value < 0.05 and a difference in IPA site usage of |ΔIPUI| > 0.1. Significant differential IPA events from APAlyzer were selected using an FDR-adjusted *p*-value < 0.05. Detailed information on the execution of these baseline methods is available in the *S1 Appendix*. As an evaluation metric, the area under the ROC curve (AUC) was used to estimate the IPA detection capacity of all methods on the simulated datasets.

## Supporting information

**Table A in S1 Appendix**. Parameters for running Flux Simulator to generate 50 million reads.
**Fig A in S1 Appendix**. Comparison of IPScan with four baseline methods for the identification of potential IPA sites with 30M reads. Performance was benchmarked against 1,000 simulated ground-truth events.
**Fig B in S1 Appendix**. Comparison of IPScan with four baseline methods for the identification of potential IPA sites with 10M reads. Performance was benchmarked against 1,000 simulated ground-truth events.
**Fig C in S1 Appendix**. Comparison of IPScan with four baseline methods for the identification of potential IPA sites with 5M reads. Performance was benchmarked against 1,000 simulated ground-truth events.
**Fig D in S1 Appendix**. Nucleotide composition around the IPA sites detected by IPScan in the MEF WT sample. The x-axis represents the position relative to the IPA sites (±50 bp), while the y-axis indicates the proportion of each nucleotide at each position.
**Fig E in S1 Appendix**. Nucleotide composition around the IPA sites detected by IPScan in the MCF7 Mock sample. The x-axis represents the position relative to the IPA sites (±50 bp), while the y-axis indicates the proportion of each nucleotide at each position.
**Fig F in S1 Appendix**. Nucleotide composition around the IPA sites detected by IPScan in the MCF7 Torin sample. The x-axis represents the position relative to the IPA sites (±50 bp), while the y-axis indicates the proportion of each nucleotide at each position.
**Fig G in S1 Appendix**. Nucleotide composition around the IPA sites detected by IPScan in the BT549 Mock sample. The x-axis represents the position relative to the IPA sites (±50 bp), while the y-axis indicates the proportion of each nucleotide at each position.
**Fig H in S1 Appendix**. Nucleotide composition around the IPA sites detected by IPScan in the BT549 Torin sample. The x-axis represents the position relative to the IPA sites (±50 bp), while the y-axis indicates the proportion of each nucleotide at each position.
**Realtime quantitative PCR (RT-qPCR) analysis and primer sequences. PepQuery run parameters. Running the baselines.**​
(PDF)
