# Supplementary material for: IPScan: Detecting novel intronic PolyAdenylation events with RNA-seq data
Source: PLoS Comput Biol. 2025 Nov 11;21(11):e1013668. doi: 10.1371/journal.pcbi.1013668 (PMC12626307; doi:10.1371/journal.pcbi.1013668)
Supplement: Supplementary file 1 — Table A in S1 Appendix. Parameters for running Flux Simulator to generate 50 million reads. Fig A in S1 Appendix. Comparison of IPScan with four baseline methods for the identification of potential IPA sites with 30M reads. Performance was benchmarked against 1,000 simulated ground-truth events. Fig B in S1 Appendix. Comparison of IPScan with four baseline methods for the identification of potential IPA sites with 10M reads. Performance was benchmarked against 1,000 simulated ground-truth events. Fig C in S1 Appendix. Comparison of IPScan with four baseline methods for the identification of potential IPA sites with 5M reads. Performance was benchmarked against 1,000 simulated ground-truth events. Fig D in S1 Appendix. Nucleotide composition around the IPA sites detected by IPScan in the MEF WT sample. The x-axis represents the position relative to the IPA sites (±50 bp), while the y-axis indicates the proportion of each nucleotide at each position. Fig E in S1 Appendix. Nucleotide composition around the IPA sites detected by IPScan in the MCF7 Mock sample. The x-axis represents the position relative to the IPA sites (±50 bp), while the y-axis indicates the proportion of each nucleotide at each position. Fig F in S1 Appendix. Nucleotide composition around the IPA sites detected by IPScan in the MCF7 Torin sample. The x-axis represents the position relative to the IPA sites (±50 bp), while the y-axis indicates the proportion of each nucleotide at each position. Fig G in S1 Appendix. Nucleotide composition around the IPA sites detected by IPScan in the BT549 Mock sample. The x-axis represents the position relative to the IPA sites (±50 bp), while the y-axis indicates the proportion of each nucleotide at each position. Fig H in S1 Appendix. Nucleotide composition around the IPA sites detected by IPScan in the BT549 Torin sample. The x-axis represents the position relative to the IPA sites (±50 bp), while the y-axis indicates the proportion of each nucleotide at each pos [file pcbi.1013668.s001.pdf]

IPScan: Detecting Novel Intronic PolyAdenylation Events with RNA-seq Data

Naima Ahmed Fahmi[1], Sze Cheng[2], Jeovani Overstreet[1], Qianqian Song[3], Jeongsik Yong[2*], Wei Zhang[1*]

1 Department of Computer Science, University of Central Florida, Orlando, Florida, United States of America;
2 Department of Biochemistry, Molecular Biology and Biophysics, University of Minnesota Twin Cities, Minneapolis, Minnesota, United States of America;
3 Department of Health Outcomes and Biomedical Informatics, University of Florida, Gainesville, Florida, United States of America.

*Emails: wzhang.cs@ucf.edu and jyong@umn.edu

Running Flux Simulator

Table A. Parameters for running Flux Simulator to generate 50 million reads.

**Parameters**	**Value**	**Description**
REF_FILE_NAME	mm10.refGene.gtf	GTF reference annotation
GEN_DIR	Genome_mm10	Genomic sequences directory
NB_MOLECULES	10000000	Number of RNA molecules
TSS_MEAN	100	
POLYA_SCALE	100	Transcript modification parameters
POLYA_SHAPE	2	
FRAG_SUBSTRATE	DNA	
FRAG_METHOD	NB	Library Preparation parameters
FRAG_NB_LAMBDA	575	
FRAG_NB_M	1	
RTRANSCRIPTION	YES	Switch on reverse transcription
PCR_DISTRIBUTION	default	
GC_MEAN	NaN	Amplification parameters
GC_SD	NaN	
PCR_PROBABILITY	0.1	
FILTERING	YES	Switches size selection On
UNIQUE_IDS	TRUE	Create Unique Read Identifiers for paired-end
READ_NUMBER	50000000	Number of reads
READ_LENGTH	76	Length of each read
PAIRED_END	YES	Paired end reads
FASTA	YES	Generate Fasta file
ERR_FILE	76	Error model for length 76

Performance comparison of the baselines with different read depths

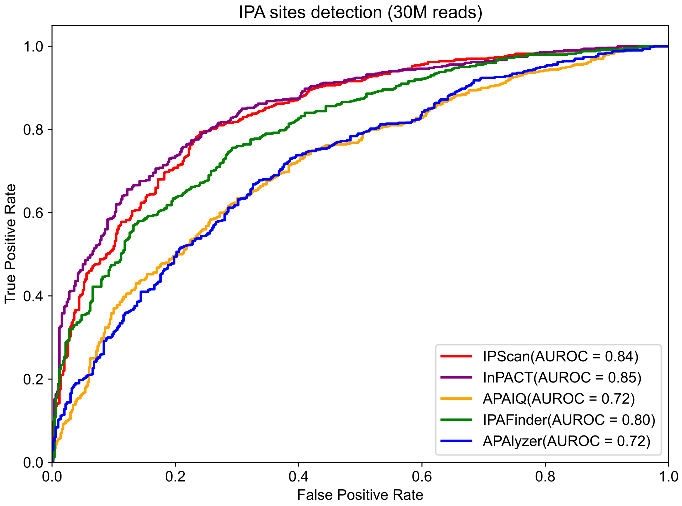

Fig A. Comparison of IPScan with four baseline methods for the identification of potential IPA sites with 30M reads. Performance was benchmarked against 1,000 simulated ground-truth events.

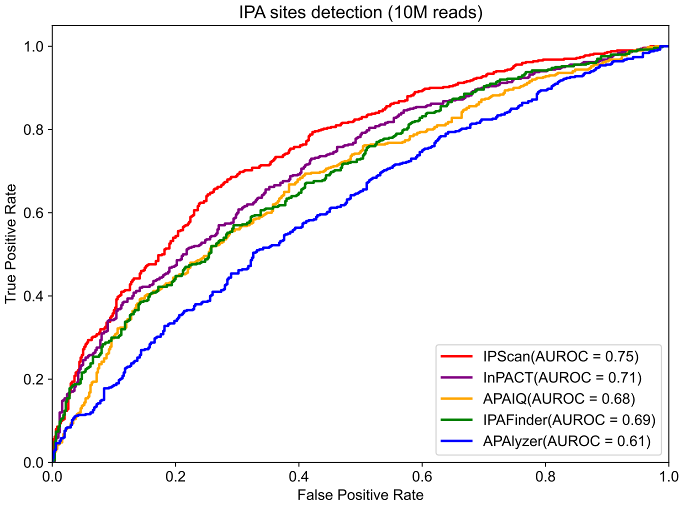

Fig B. Comparison of IPScan with four baseline methods for the identification of potential IPA sites with 10M reads. Performance was benchmarked against 1,000 simulated ground-truth events.

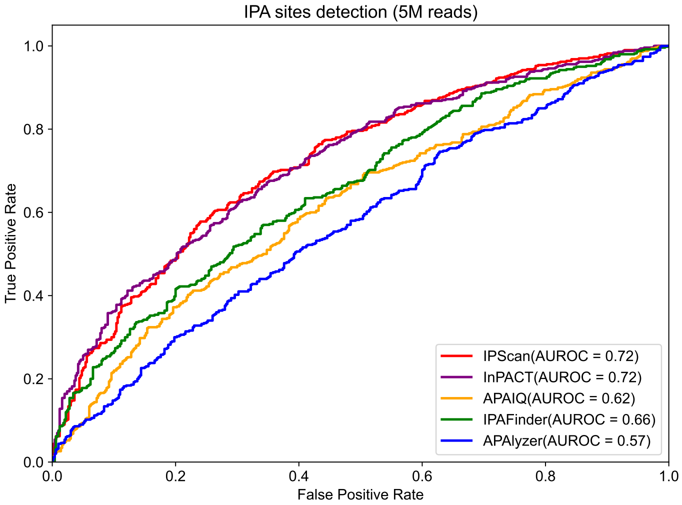

Fig C. Comparison of IPScan with four baseline methods for the identification of potential IPA sites with 5M reads. Performance was benchmarked against 1,000 simulated ground-truth events.

Nucleotide composition near the IPA sites on different cell line data

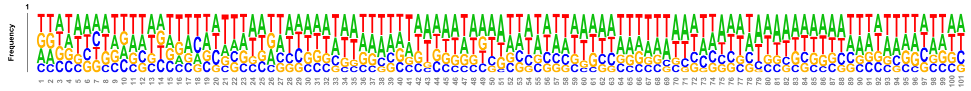

Fig D. Nucleotide composition around the IPA sites detected by IPScan in the MEF WT sample. The x-axis represents the position relative to the IPA sites (±50 bp), while the y-axis indicates the proportion of each nucleotide at each position.

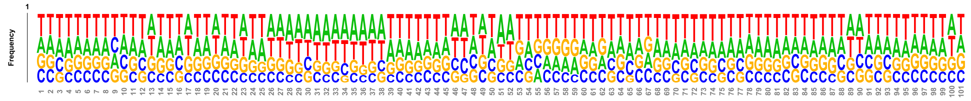

Fig E. Nucleotide composition around the IPA sites detected by IPScan in the MCF7 Mock sample. The x-axis represents the position relative to the IPA sites (±50 bp), while the y-axis indicates the proportion of each nucleotide at each position.

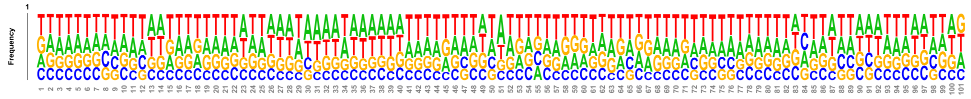

Fig F. Nucleotide composition around the IPA sites detected by IPScan in the MCF7 Torin sample. The x-axis represents the position relative to the IPA sites (±50 bp), while the y-axis indicates the proportion of each nucleotide at each position.

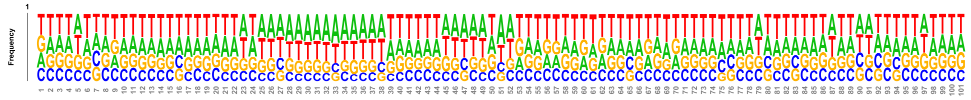

Fig G. Nucleotide composition around the IPA sites detected by IPScan in the BT549 Mock sample. The x-axis represents the position relative to the IPA sites (±50 bp), while the y-axis indicates the proportion of each nucleotide at each position.

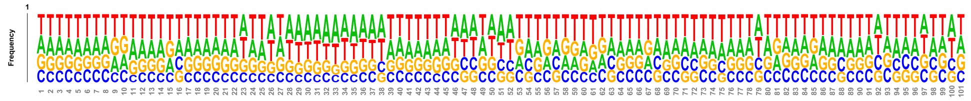

Fig H. Nucleotide composition around the IPA sites detected by IPScan in the BT549 Torin sample. The x-axis represents the position relative to the IPA sites (±50 bp), while the y-axis indicates the proportion of each nucleotide at each position.

Realtime quantitative PCR (RT-qPCR) analysis and primer sequences

Realtime quantitative PCR (RT-qPCR) analysis and primer sequences: Total RNAs from WT MEFs and Tsc1-/- MEFs were isolated using TRIzol reagents (Thermo Fisher Scientific) according to manufacturer's protocol (https://assets.thermofisher.com/TFS-Assets/LSG/manuals/trizol_reagent.pdf). cDNAs were synthesized by reverse transcription using Oligo-dT priming and NxGen M-MuLV Reverse Transcriptase (LGC Biosearch Technologies) according to the manufacturer's protocol (https://www.lucigen.com/docs/manuals/MA115-M-MuLV.pdf) and used for quantitative PCR. The Ct values were quantified in real time using EvaGreen (Biotium) and the relative expression between samples was normalized with the $\Delta\Delta$Ct method.

The list of primers used for qRT-PCR includes:

Map3k10 forward: 5'-GATGACCTTCGGACCAAGGAG-3'

Map3k10 reverse: 5'-GGAGGGACCAGGGAAATTCTG-3'

Troap forward: 5'-CTGGGGGATCAAATGTGGGG-3'

Troap reverse: 5'-GCAGTATGCAAGACCTCCACT-3'

PepQuery Run parameters

Databases:

CPTAC_TCGA_Breast_Cancer_Proteome_PDC000173

CPTAC_TCGA_Breast_Cancer_Phosphoproteome_PDC000174

Command:

java -jar pepquery-2.0.2.jar -t pep -s 1 -maxLength 100 -fast -b <databases> -db RefSeq:human -hc -o <Output_dir> -i <input_sequence_file>

Output parameters:

Confident='yes'

P-value cutoff <= 0.01

n_ptm==0

Running the baselines

IPAFinder

Command for IPA sites detection in single sample:
python3 IPAFinder_DetectIPA.py -b sample1.bam -anno annotation.txt -p 10 -o sample_50M_IPA.txt

Command for differential IPA detection (with replicates):
Rscript Infer_DUIPA.R -b bamfiles_list.txt -I IPAFinder_IPUI.txt -d 50M_case_control_dir -o IPAFinder_DUIPA.txt

APAlyzer

Download and prepare annotated IPA sites (R commands):
URL=”https://github.com/RJWANGbioinfo/PAS_reference_RData/blob/master/”
file=”mm10_REF.RData”
source_data(paste0(URL,file,”?raw=True”)
PASREF=REF4PAS(refUTR,dfIPA,dfLE)
dfIPA=PASREF$dfIPA
dfLE=PASREF$dfLE

Calculation of relative expression of the IPA sites (R commands):
IPA_OUT=PASEXP_IPA(dfIPA, dfLE, flsall, Strandtype=”forward”, nts=1)

Find significantly regulated IPA in two groups (R commands):
samples_list = data.frame(samplename = c(names(flsall)), condition = c(rep(”control”,3),rep(”case”,3)))
IPA_diff=APAdiff(samples_list, IPA_OUT, conKEY=’control’, trtKEY=’case’, PAS=’IPA’, CUTreads=0)

InPACT

As InPACT doesnt provide IPA sites for any species other than human (hg38), we have constructed the InPACT_polyAsites.mm10.saf file for mouse(mm10) using PolyA_DB (v3) with the recommended format shown below and by selecting regions from 20 nt upstream to the poly(A) site:

GeneID	Chr	Start	End	Strand
chr1:OR4F5	chr1	65451	65470	+
chr1:OR4F5	chr1	65482	65501	+
chr1:OR4F5	chr1	65757	65776	+

Command to identify the IPA sites
InPACT -i sample.bam -a RefSeq_mm10.gtf -s InPACT_polyAsites.mm10.saf -P 5

Command to assemble novel transcripts
InPACT_transcript
–predict_terminal predict.result.txt
–annotated_gtf RefSeq_mm10.gtf
–fa_path mm10.fa
–save_gtf merged.gtf

Commands to quantify and compute IPA usage

1. *gffread merged.gtf -g mm10.fa -w transcripts.fa*
2. *salmon index -t transcripts.fa -i transcripts_ex -k 31*
3. *salmon quant -i transcripts_index -l A -1 reads_1.fastq.gz -2 reads_2.fastq.gz*
   –validateMappings -o transcripts_quant
4. *InPACT_quantify*
   –transcript_tpm transcripts_quant/quant.sf
   –annotation_file merged.gtf
   –ipa_info predict.result.txt
   –save_file ipa_usage.txt

APAIQ

APAIQ requires a poly(A) reference (BED) to annotate hits and compute distances. For mouse mm10, we used PolyA_DB (v3) and converted it to BED (1-bp PAS).

Commands to identify PAS

1. *apaiq –input_file sample.mm10.RPM.bedGraph*
   –out_dir out_mm10
   –fa_file mm10.fa
   –name sample1
   –DB_file polyA.mm10.rep.bed
   –model snu398_model.ckpt
2. *awk 'NR==1 || $4 >=12' out_mm10/sample1.out.txt > sample1.PAS.score12.txt*
3. *bedtools intersect -u -a sample1.PAS.score12.bed -b introns.mm10.bed >*
   IPA.mm10.predicted.bed

Command to quantify PAU for the IPA sites

python regression/evaluateRegression.v.2.py
–model regression/regression.ckpt
–factor_path regression/normalize_factor
–pas_file IPA.mm10.predicted.bed
–input_file sample.mm10.RPM.bedGraph
–out IPA.mm10.PAU.txt
–threshold 0
